# Allergen Immunotherapy–Induced Immunoglobulin G4 Reduces Basophil Activation in House Dust Mite–Allergic Asthma Patients

**DOI:** 10.3389/fcell.2020.00030

**Published:** 2020-02-20

**Authors:** Mulin Feng, Xiaohui Zeng, Qiujuan Su, Xu Shi, Mo Xian, Rundong Qin, Jing Li

**Affiliations:** Department of Allergy and Clinical Immunology, Guangzhou Institute of Respiratory Health, State Key Laboratory of Respiratory Disease, The First Affiliated Hospital of Guangzhou Medical University, Guangzhou, China

**Keywords:** asthma, rhinitis, allergen immunotherapy, basophil activation test, serum specific immunoglobulin G4 (IgG4)

## Abstract

It is unclear if allergen immunotherapy (AIT) can reduce allergy effector cell activation. We evaluated the basophil response during *Dermatophagoides pteronyssinus* (Der p) subcutaneous immunotherapy (SCIT) and its relationship to allergen-specific immunoglobulin G4 (sIgG4) in allergic rhinitis and/or asthma patients. The study included 55 subjects, of which 35 cases received Der p SCIT and 20 controls received standard medications. Symptom and medication scores (SMSs), sIgG4 levels, specific immunoglobulin E (sIgE) levels, allergen-induced basophil activation tests (BATs) in whole blood, and BAT inhibition assays in serum were determined at weeks 0, 4, 12, 16, 52, and 104 of SCIT. Levels of Der p sIgG4 in SCIT patients significantly increased after 12 weeks of treatment compared to week 0. Serum obtained from SCIT patients significantly inhibited basophil activation after 12 weeks of treatment. Removal of immunoglobulin G4 (IgG4) antibodies at week 104 reduced the ability of serum to block basophil activation. An increase of Der p sIgG4 rather than reduction of Der p sIgE correlated with the reduction of basophil activation during SCIT. The sIgG4 antibodies may compete with sIgE binding to allergens to form an immunoglobulin E (IgE)–allergen complex. SCIT reduced the sensitivity of allergen-triggered basophil activation in Der p allergic rhinitis and/or asthma patients through induction of sIgG4.

## Introduction

Allergen-specific immunotherapy (AIT) is an effective treatment for many allergens including house dust mite allergens (Wang et al., 2006; Durham et al., 2012; Nelson, 2014), which are a major cause of allergic rhinitis and allergic asthma in China (Li et al., 2009). The therapeutic mechanisms of AIT involve modulation of cellular reactions and related antibody responses as well as inhibition of anaphylactic cell release of their mediators (Till et al., 2004).

Cellular changes include generation of allergen-specific regulatory subsets of T and B cells and inhibition of allergen-specific T helper type 2 (Th2) cells (Wachholz et al., 2002). Antibody responses are involved in the induction of allergen-specific immunoglobulin G (IgG) antibodies, in particular, IgG4 antibodies. IgG4 antibodies may have blocking activities, as they compete with specific immunoglobulin E (sIgE) for binding to the allergen. This inhibits allergen–immunoglobulin E (IgE) complex formation on sIgE receptor–expressing cells such as mast cells and basophils. The responses of basophils to allergen stimulation are dose dependent. *Reactivity* is defined as the maximal response plateau to allergen stimulation located on the dose-response curve. Cellular *sensitivity* represents the threshold of allergen sensitivity under submaximal allergen concentration stimulation (MacGlashan, 1993). The basophil activation test (BAT) is a useful tool for allergy diagnosis (Gonzalez-Munoz et al., 2008; Santos et al., 2014; Imoto et al., 2015); studies have provided evidence for its usefulness in monitoring the induction of immune tolerance by immunotherapy (Bidad et al., 2014; Kepil Ozdemir et al., 2014). Potapinska et al. demonstrated that BAT has high sensitivity and specificity values in the diagnosis of atopic diseases (Potapinska et al., 2009; Ozdemir et al., 2011). In a peanut immunotherapy study, Santos et al. found that depletion of IgG4 reduced the inhibitory effect of peanut-induced basophil activation (Santos et al., 2015). Our previous study demonstrated that IgG4 responses are prominent during *Dermatophagoides pteronyssinus* (Der p) subcutaneous immunotherapy (SCIT) (Lai et al., 2013). In this study, we investigated the relationship between basophil response and IgG4 antibodies to demonstrate that Der p SCIT might reduce basophil reactivity and/or sensitivity through induction of IgG4 in dust mite–sensitive subjects.

## Materials and Methods

### Study Design and Population

The study included a total of 55 subjects, 21 children (age ≤ 14 years) and 34 adults (age 15–57 years), with mild-to-moderate asthma and/or rhinitis. Of these patients, 35 cases received Der p SCIT, and 20 controls received regular medications, serving as the medication group (Table 1). All patients came from the allergy and clinical immunology department of the Guangzhou Institute of Respiratory Diseases, fulfilled the ARIA guideline for allergic rhinitis and/or GINA guideline for mild-to-moderate asthma (Bousquet et al., 2007, 2008), and had a positive skin prick test (SPT) and sIgE to Der p ≥ 0.7 kU/L (ImmunoCap, Pharmacia, Sweden). All patients had a forced vital capacity in the first second (FEV_1_) greater than 70% of the predicted value before enrolling into the study. Patients visited the hospital for treatments and clinical evaluations. Serum samples were collected before initiation of SCIT and at weeks 4, 12, 16, 52, and 104 during the treatment. The study protocol was approved by the Ethics Committee of the First Affiliated Hospital of Guangzhou Medical University and registered at http://www.chictr.org.cn (ChiCTR-OOC-15006207). Written informed consent was obtained from all adult patients or the parents of children. We estimated an appropriate sample size based on our data focusing on SCIT patients and containing paired samples and independent samples. We used a one-tailed test for independent samples with the alpha level set at 0.05 and the effect size equal to 0.8, with a statistical power of 0.90. The allocation ratio of the SCIT group to the medication group was 1.5. The estimation indicated that at least 35 individuals in the SCIT group and 23 individuals in the medication group would be required.

**TABLE 1:** Baseline information for patients in SCIT and medication groups.

Items	SCIT group	Medication group	*P* Value
	(*n* = 35)	(*n* = 20)	
Gender m/f (cases)	25/10	12/8	>0.05
Age distribution (y)	20.3 ± 2.2	22.3 ± 2.5	>0.05
Children (≤14)	15 (9.1 ± 0.5)	6 (8 ± 0.6)	>0.05
Adults (>14)	20 (28.8 ± 2.6)	14 (30 ± 1.9)	>0.05
**Case distribution cases (%)**			
Asthma	5 (14.3)	2 (10.0)	>0.05
Rhinitis	4 (11.4)	3 (15.0)	>0.05
Asthma combined	26 (74.3)	15 (75.0)	>0.05
**Rhinitis**			
Symptom and medication	3.67 ± 0.42	3.82 ± 0.45	>0.05
score			
FEV_1_ (%predicted)	91.16 ± 2.25	91.64 ± 1.81	>0.05
FVC (%predicted)	98.05 ± 1.50	93.85 ± 2.17	>0.05
Total IgE (kU/1)	477 ± 65	562 ± 47	>0.05
Specific IgE to Der p (kU/1)	85 ± 11	87 ± 11	>0.05

*Data are presented as mean ± SE. FEV_1_, forced expiratory volume at 1s; FVC, forced vital capacity; IgE, immunoglobulin E.*

### Detection of Serum IgE and IgG4

The levels of total IgE and sIgE against Der p were measured by a Pharmacia CAP fluorescence enzyme immunoassay system (ThermoFisher, Sweden). The sIgE results are reported as kU/L, with a lower limit of 0.35 kU/L and an upper detection limit of 100 kU/L. Serum Der p allergen-specific IgG4 (sIgG4) levels were measured using a four-layer sandwich enzyme-linked immunosorbent assay (ELISA) system as previously reported (Lai et al., 2013).

### SCIT Protocol

The patients were treated with subcutaneous injections of standardized aluminum-formulated Der p Alutard-SQ vaccine (ALK-Abello A/S, Horsholm, Denmark). The treatment protocol followed the recommended up-dosing schedule of 16 weeks before reaching a maintenance dose of 100,000 Alutard-SQ, for a duration of 2 years of SCIT.

### Clinical Evaluations

The patients were asked to complete a symptom and medication diary routinely during the whole course of treatment. Patients were asked to rate symptoms of asthma (daytime: 0–5; nighttime: 0–4) and rhinitis (day- or nighttime: 0–2) according to the severity and frequency of the symptoms in disturbing daily activities and sleep (Wang et al., 2006). The daily medication score was calculated by assigning a score of 1 for each 160 μg of budesonide or the equivalent dose of inhaled corticosteroid, or each 130 μg of budesonide or the equivalent dose of nasal corticosteroid, as well as for each puff of salbutamol/terbutaline or the equivalent dose of another inhaled β2-agonist and for each 10 mg of oral loratidine or the equivalent dose of another anti-histamine tablet. A symptom and medication score (SMS) was defined as the sum of symptom scores and medication scores (Canonica et al., 2007).

### Evaluation of Basophil Activation via Measurement of CD63 Expression

Basophil activation tests were performed as reported in Santos et al. (2014) at weeks 0, 4, 12, 16, 52, and 104. First, we performed dose-finding experiments in 10 HDM-allergic patients and 5 non-atopic controls. Heparinized whole blood (100 μl) was stimulated for 30 min at 37°C with Der p extract (ALK-Abello A/S, Horsholm, Denmark) diluted in phosphate-buffered saline (PBS, Sigma Diagnostics, St. Louis) at serial sixfold dilutions (150, 15, 1.5, 1.5 × 10^–1^, 1.5 × 10^–2^, 1.5 × 10^–3^ μg/ml). The two allergen concentrations that evoked maximal (i.e., 15 μg/ml) and submaximal (i.e., 0.15 μg/ml) cell stimulation were chosen for the study to investigate the time course of BAT during the SCIT. Before erythrocyte lysis, cells were stained with CD123-PE-Cy5 (BD), CD203c-PE, HLA-DR-ECD, and CD63-FITC (Beckman Coulter). Basophils gated as SSC^low^/CD203c^+^/CD123^+^/HLA-DR^–^ were detected by flow cytometry (Beckman Coulter Epics XL-MCL, United States) and analyzed using FCS Express software (version 4).

### Basophil Activation Inhibition Assays

Heparinized whole venous blood was obtained from three HDM atopic adult volunteers. The sIgE values for Der p were 90, 104, and 159 kU/L, respectively. The percentage of CD63 was pre-determined by the BAT as previously described (the basic basophil activation is 22, 25, and 26% at 0.15 μg/ml Der p, and 62, 63, and 65% at 15 μg/ml Der p, respectively). Serum (10 μl) from SCIT patients or medication subjects was incubated with 30 μl of Der p allergen (the final concentration of Der p was 0.15 or 15 μg/ml) at 37°C for 1 h. We then added 100 μl of HDM atopic donor blood and incubated the sample at 37°C for 30 min. The following steps were performed as BAT (i.e., stained with antibodies and detected by flow cytometry).

### IgG4 Antibody Depletion and Retest of BAT Inhibition Assays

IgG1 anti-IgG4 antibody (Fitzgerald) was coupled to cyanogen bromide (CNBr)–activated Sepharose (GE Healthcare, Hertfordshire, United Kingdom) as described by Kavran and Leahy (2014). Mock-coupled Sepharose beads were prepared and incubated with coupling buffer lacking antibody to generate a negative control. The following depletion steps were performed as described by Santos et al. (2015). Briefly, the remaining reactive CNBr sites were blocked with 1 mol/L ethanolamine and then washed in alternating pH using 0.1 mol/L acetic acid/sodium acetate at pH 4.0 and 0.1 mol/L Tris–HCl at pH 8.0 for three cycles. Fifteen serum samples from patients after 104 weeks of SCIT, with the largest ratio of basophil CD63 expression at week 0 and week 104 according to the BAT inhibition assay results, were diluted 1:5 in PBS-AT (0.3% BSA, 0.1% Tween 20, and 0.05% NaN3 in PBS). Diluted serum samples were incubated with anti-IgG4- or mock-coupled Sepharose beads overnight and collected by means of centrifugation. The specificity of the depletion of Der p specific antibody was confirmed by a four-layer sandwich ELISA as previously demonstrated (Lai et al., 2013). After IgG4 antibody depletion, samples were assayed for BAT inhibition assays as previously described. The percentage of inhibition calculation formula was as follows:

%inhibition

 =(%CD63cellssensitizedwithserumfromanHDM

atopicvolunteerand0.15μg/mlDerp-%CD63cells

sensitizedwithtestserumand0.15μg/mlDerp)

/(%CD63cellssensitizedwithserumfromanHDM

atopicvolunteerand0.15μg/mlDerp).

### Statistical Analysis

An independent-samples *t*-test was used to analyze group differences in SMS, Der p sIgG4, BAT, and BAT inhibition assays. A paired-samples *t*-test was used to analyze within-group differences. Data are presented as mean ± SE. Linear regression was employed to analyze the relationship between Der p sIgG4, Der p sIgE, and BAT inhibition assays. Differences were considered significant at *P* < 0.05.

## Results

### Patient Characteristics

The demographic data, SMS, and antibody levels of all subjects are shown in Table 1. There were no differences between SCIT and medication group in gender, SMSs, and serum IgE levels.

### Changes of Clinical Outcomes

The combined symptom medication score (SMS) of asthma and rhinitis decreased significantly after 12 weeks of treatments compared to baselines in both groups (Supplementary Figure S1A), with more significant declines seen in the SCIT subjects compared to medication-treated subjects at weeks 52 and 104 (Supplementary Figure S1A). FEV_1_% did not change significantly during treatment in either group (Supplementary Figure S1B).

### Allergen Concentration Curves for BAT

Basophil activation in mite allergy patients (*n* = 10) and non-atopic controls (*n* = 5) was assayed with six mite extract concentrations. The analysis of allergen concentration curves for the BAT showed no significant change in CD63 in non-atopic controls (*P* > 0.05). In contrast, BAT results were dose dependent in the mite-allergic patients (Supplementary Figure S1C). The two allergen concentrations that evoked maximal (i.e., 15 μg/ml) and submaximal (i.e., 0.15 μg/ml) cell stimulation were chosen to further investigate the time course of basophil activation.

### Time Course of Basophil Activation During SCIT

**FIGURE 1:**
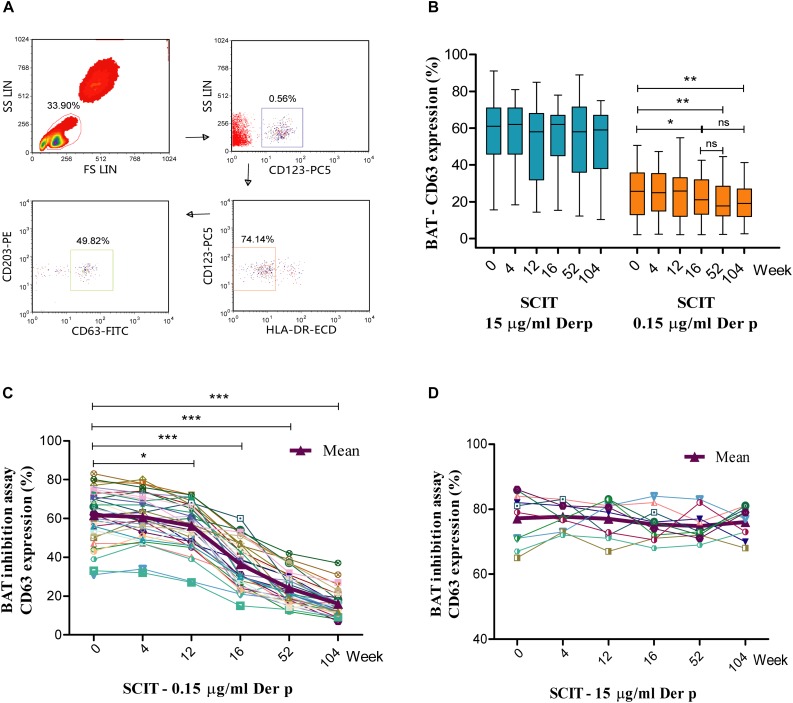
Time course of basophil activation test (BAT) and BAT inhibition assay during subcutaneous immunotherapy (SCIT). Lymphocytes are identified by their scatter properties, and the activated basophils gated as SSC^low^/CD203c^+^/CD123^+^/HLA-DR^–^ **(A)**. The basophil CD63 responses to 0.15 and 15 μg/ml of Der p extract during the time course of BAT **(B)**. The central box represents the values from the lower to the upper quartile (25th and 75th percentiles); the line within the box indicates the median, and the whiskers show the 5th and 95th percentiles. The BAT inhibition assay was performed with serum from SCIT patients incubated with 0.15 μg/ml (**C**; *n* = 35) or 15 μg/ml (**D**; *n* = 10) Der p allergen before performance of the BAT. Der p = *Dermatophagoides pteronyssinus*. *P < 0.05, **P < 0.01, ***P < 0.001 when compared with week 0; ns = non-significant.

Activated basophils gated as SSC^low^/CD203c^+^/CD123^+^/HLA-DR^–^ (Figure 1A). Basophil activation responses to the 0.15 μg/ml allergen concentrations decreased significantly in the SCIT group from week 16 to 104 (16 weeks = 21.5%, *P* = 0.023; 52 weeks = 19.7%, *P* = 0.009; 104 weeks = 20.0%, *P* = 0.001) compared to baseline (0 week = 25.6%, Figure 1B). However, basophil activation was not significantly changed after stimulation with 15 μg/ml of allergen extracts at the six time points in course of SCIT (*P* > 0.05, Figure 1B). In the medication group, no significant changes were observed at the six time points in the basophil CD63 response to 15 and 0.15 μg/ml of allergen extract compared to baseline (*P* > 0.05, Supplementary Figure S2A).

### Time Course of the BAT Inhibition Assay

Serum obtained from SCIT patients significantly increased the capacity to inhibit basophil activation upon challenge with 0.15 μg/ml Der p allergen, starting from week 12 (0 week = 61.6 ± 2.3%, 4 weeks = 60.7 ± 2.0%, 12 weeks = 56.2 ± 1.9%, 16 weeks = 36.4 ± 2.0%, 52 weeks = 24.0 ± 1.4%, 104 weeks = 16.0 ± 1.1%; Figure 1C). This effect was not found when basophils were challenged with 15 μg/ml Der p allergen (Figure 1D). Serum from medication subjects did not alter basophil responses to either 0.15 or 15 μg/ml Der p allergen (Supplementary Figures S2C,D).

### Changes of Serum Der p sIgG4 and Der p sIgE Antibodies

**FIGURE 2:**
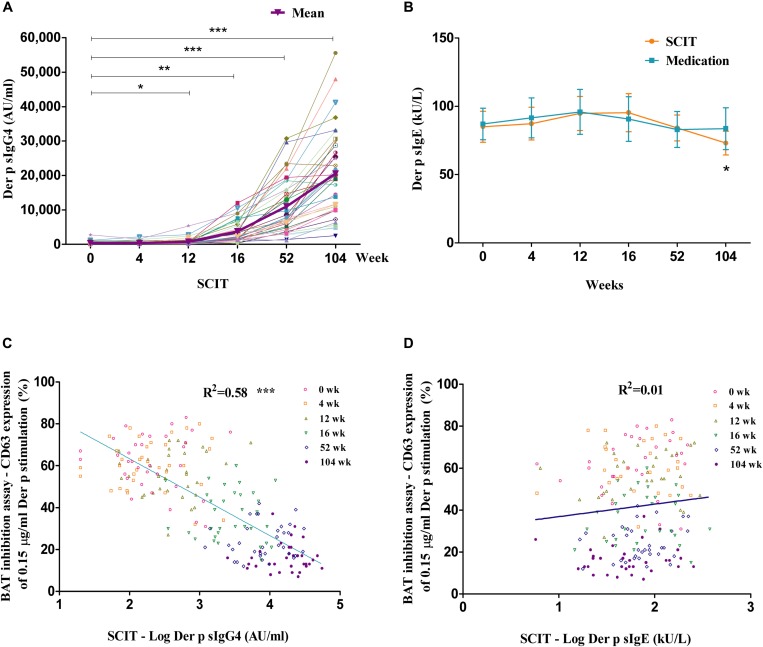
Time course of allergen-specific IgG4 (sIgG4) and specific IgE (sIgE), and the relationship with BAT inhibition assay. Time course of Der p sIgG4 **(A)** and Der p sIgE **(B)** in SCIT group. Linear regression between Der p sIgG4 and BAT inhibition assay in the SCIT group at all time points during the 2 years of treatment **(C)** and the relationship between BAT inhibition assay and Der p sIgE **(D)**. The *x*-axis is a log scale. IgG = immunoglobulin G; IgE = immunoglobulin E. *P < 0.05, **P < 0.01, ***P < 0.001 when compared with week 0.

Der p sIgG4 levels significantly increased in SCIT patients starting from week 12, with 10- to 71-fold increases from week 16 to week 104 (Figure 2A). No significant changes were observed in the medication group (Supplementary Figure S2B). The difference between the two groups was significant starting from week 16 (data not shown). Significantly lower levels of Der p sIgE were observed at week 104 in SCIT subjects (Der p sIgE: 0 week = 85 ± 11 kU/L, 4 weeks = 87 ± 12 kU/L, 12 weeks = 94 ± 12 kU/L, 16 weeks = 95 ± 14 kU/L, 52 weeks = 84 ± 9 kU/L, 104 weeks = 73 ± 9 kU/L) when compared to baseline (*P* < 0.05) (Figure 2B). No significant differences were seen in the medication group (*P* > 0.05) (Figure 2B).

### Removal of IgG4 Antibodies Reduced the Ability of Serum to Block Basophil Activation

The ability to block basophil activation was reduced after IgG4 depletion from the serum of SCIT patients at week 104 (median inhibition, mock depleted = 57.1%, IgG4 depleted = 27.9%, *P* < 0.001, *n* = 15) (Supplementary Figure S1D).

### Correlations Between sIgG4, sIgE, and BAT Inhibition Assay

Der p sIgG4 had a significant linear association with the basophil activation inhibition assay in the SCIT group at all time points during the 2 years of treatment (*R*^2^ = 0.58, *P* < 0.001; Figure 2C). There was no correlation between Der p sIgE levels and the BAT inhibition assay in the SCIT group at all time points during the 2 years of treatment (*R*^2^ = 0.01, *P* > 0.05; Figure 2D).

## Discussion

In this study of SCIT with Der p extract, we found that SCIT could significantly improve asthma symptoms and reduce medication requirements starting after 12 weeks of treatment. We demonstrated that allergen-induced basophil activation decreased after 16 weeks of AIT at submaximal allergen concentrations compared to week 0. SCIT-induced sIgG4 antibodies substantially increased, and serum obtained from SCIT patients significantly inhibited basophil activation after 12 weeks of treatment compared to week 0. Removal of IgG4 antibodies reduced the ability of blocking basophil activation, and Der p sIgG4 and basophil activation inhibition assays had a significant relationship according to linear regression analysis.

Although it was impracticable to perform a double-blind, placebo-controlled study in this 2-year clinical observation, the current investigation is consistent with a previous study (Wang et al., 2006; Lai et al., 2013) and other studies (Keles et al., 2011; Devillier et al., 2016), which confirmed that HDM AIT is an effective treatment for allergic disease.

Induction of sIgG4 has long been regarded as a prominent immunological change induced by AIT (Sahin et al., 2016). In agreement with our previous findings and those of other studies (Wachholz et al., 2003; Shamji et al., 2012; Lai et al., 2013), we found that successful AIT could induce a substantial increase of sIgG4. The magnitude of increase in Der p sIgG4 concentrations is associated with the allergen concentration used for immunotherapy (Van Metre et al., 1980). Specific IgG4 has been proposed to block antibodies by competing with sIgE for allergens to form IgE–allergen complexes. This reduces the complex binding to sIgE receptor–expressing effector cells (Gehlhar et al., 1999; Francis et al., 2008) and prevents the allergen-dependent activation of T cells by interfering with IgE-facilitated antigen presentation (Van Neerven et al., 2006). IgG antibodies have also been shown to be associated with the inhibition of allergen-induced effector cell activation (Ball et al., 1999; Mothes et al., 2003) or with reduced allergen sensitivity (Reisinger et al., 2005). Although induction of allergen-specific serum IgG is regarded as a characteristic feature of the immunological response induced by AIT, treatment-induced changes in the levels of sIgE secretion remain a controversial issue. We found that the levels of Der p sIgE decreased significantly after 104 weeks of SCIT; this is supported by other studies (Arlian et al., 1993; Chen et al., 2017; Feng et al., 2018), However, other studies demonstrated that sIgE levels did not change after 1 year of AIT (Shamji et al., 2011; Sahin et al., 2016), and Blumberga et al. (2011) found that AIT-induced serum sIgE increased initially and then declined to baseline value after 1 year of treatment. In general, the decrease of sIgE during the late phase of AIT might be associated with the secretion of IL-10 and TGF-β by Treg cells as well as the switching of allergen-specific B cells toward IgG4 production instead of sIgE production (Frew, 2010).

We found that basophil activation triggered by submaximal allergen concentrations decreased after 16 weeks of AIT treatment. However, basophil activation was not significantly changed with maximal allergen concentrations in either the AIT or the medication group. This observation is consistent with other studies (Ebo et al., 2007; Lalek et al., 2010; Kepil Ozdemir et al., 2014), showing that basophil activation decrement was observed mainly in submaximal allergen stimulation in BAT experiments. However, in some studies, allergen-induced basophil CD203c expression did not change after 4 months of grass pollen sublingual immunotherapy (Horak et al., 2009). Erdmann et al. (2004) did not observe a change in basophil activation after 6 months of venom immunotherapy. Differences in the type of allergy, BAT markers used, time course of immunotherapy, and the allergen stimulation concentration may help explain the contradicting results in different studies.

This is the first study to assess the blocking function of serum IgG antibodies during HDM SCIT in allergic rhinitis and/or asthma patients by using the basophil activation inhibition assay. Studies have found that specific IgG4s have blocking activities by competing with sIgE for allergens to form allergen–IgE complexes. This inhibits complex binding to IgE receptor–expressing effector cells by means of the IgE-facilitated allergen binding assay (Wachholz et al., 2003; Shamji et al., 2012; Feng et al., 2018). These studies demonstrated that IgG antibodies could inhibit the allergen–IgE complex binding to the low-affinity IgE receptor (FcεRII, i.e., CD23) by use of a CD23-expressing Epstein–Barr virus-transformed B cell line and may thereby reduce allergen-specific T cell responses. We found that the serum obtained from SCIT patients significantly inhibited basophil activation and that basophils express high-affinity IgE receptor (FcεRI). Therefore, we demonstrated the effectiveness of IgG blocking activities in the high-affinity IgE receptor–expressing effector cell. We also found that the expression of CD63 in the basophil activation inhibition control (i.e., week 0) was higher than the basic basophil activation, and the Der p sIgE level significantly decreased at the late phase of SCIT. However, there was no correlation between the BAT inhibition assay and sIgE. The IgE-sensitized basophils may have already bound with IgE on the cell surface FcεRI, so that the FcεRI-bound IgE rather than serum free sIgE plays a more important role in the BAT inhibition assay.

Since the reduction of BAT appeared associated with the concentration of the triggering allergen, we considered the likely involvement of inhibiting antibodies. The inhibition experiments showed that serum from SCIT patients reduced basophil allergen threshold sensitivity but had no effect on basophil reactivity. We also depleted IgG4 from the serum of patients after completion of 2 years of SCIT and found that the IgG4-deficient serum demonstrated reduced inhibitory effects on basophil activation. Linear regression showed significant correlations between sIgG4 and the basophil activation inhibition assay. Thus, IgG4 antibodies appear to play an important role in reducing allergen sensitivity rather than reducing reactivity during AIT. IgG4 depletion could not remove the inhibition ability completely; the existence of other antibody isotypes such as IgA and IgG1 may also have inhibition ability (Santos et al., 2015). Further investigation on these antibody isotypes may help explain this phenomenon.

IgG4 is a unique antibody with a half-antibody exchange; it is also referred to as “Fab-arm exchange,” which results in monovalent and non-crosslinking antibodies (Aalberse et al., 2009). IgG4 antibodies can bind to allergens and reduce the free allergen concentration but not induce effector cell activation. Our results are consistent with other studies. For example, Nopp et al. (2009) observed negative correlations between basophil activation and specific IgG antibodies (20-fold median increase of sIgG4 after 9 months of SCIT). Lalek et al. (2010) showed that birch-specific IgG antibodies are responsible for the reduction of basophil allergen threshold sensitivity (fivefold median increase of sIgG4 after 2–4 months of SCIT). We reported 10-fold increases of sIgG4 after 4 months of SCIT. However, Horak et al. (2009) demonstrated that basophil responsiveness did not change after 4 months of grass sublingual immunotherapy and only found a twofold median increase in sIgG4 antibodies. These results may also explain why AIT had no or less effect on basophil reactivity (Lalek et al., 2010), as there were not sufficient antibodies to compete with IgE binding to the allergen.

Previous studies demonstrated that AIT could induce a substantial increase of sIgG4 antibodies and had blocking activity, but allergen-specific IgG does not always correlate with therapeutic effects (Bodtger et al., 2005). This could be explained by AIT not only increasing the level of serum IgG4 antibodies but also altering their specificity and/or affinity. Many studies have demonstrated that AIT not only increases the level of serum IgG4 antibodies but also alters their specificity (Michils et al., 1997, 1998). In venom immunotherapy, Michils et al. (1997) found that specificity change can occur at the beginning of treatment and long before the change of antibody titers could be detected. Kolbe et al. (1995) showed that administration of high concentrations of allergen in murine models alter not only antibody quantity but also affinity and specificity. However, Wachholz et al. (2003) showed that the binding affinities of allergen-specific IgG or IgE did not change after immunotherapy. Thus, the relationship between IgG4 and clinical efficacy requires further investigation.

## Conclusion

We demonstrated the time course of both the cellular and humoral immune responses during HDM immunotherapy, including the induction of sIgG4 antibodies and reduction of basophil sensitivity to the allergen. We also demonstrated that an increase of Der p sIgG4 rather than a reduction of Der p sIgE correlates with the reduction of basophil activation. SCIT reduced the allergen basophil threshold sensitivity in Der p allergic rhinitis and/or asthma patients through induction of sIgG4.

## Data Availability Statement

All datasets generated for this study are included in the article/Supplementary Material.

## Ethics Statement

The studies involving human participants were reviewed and approved by the Ethics Committee of the First Affiliated Hospital of Guangzhou Medical University. Written informed consent to participate in this study was provided by the participants’ legal guardian/next of kin.

## Author Contributions

JL designed the study, collected the data, and performed the statistical analysis. MF had responsibility for the experiments, performed the statistical analysis, and drafted the manuscript. XZ and RQ were involved in statistical analysis. QS, MX, and XS were involved in data collection.

## Conflict of Interest

The authors declare that the research was conducted in the absence of any commercial or financial relationships that could be construed as a potential conflict of interest.

## Supplementary Material

FIGURE S1 SMS and FEV1% over time. Allergen concentration curves for BAT, and IgG4 depletion BAT inhibition assay. Time course of mean SMS **(A)** and FEV_1_% predicted **(B)** in the SCIT group and medication group. Der p induced basophil activation in blood samples from allergic patients with mite allergy (*n* = 10), and non-atopic controls (*n* = 5) were assayed with six mite extract concentrations. Results are expressed as percentages of CD63^+^ basophils above basal values **(C)**. Fifteen serum samples from SCIT patients at week 104 underwent removal of IgG4 antibodies. IgG4-depleted and mock-depleted samples were incubated with 0.15 μg/mL Der p allergen before performance of the BAT. **P* < 0.05, ***P* < 0.01 when compared with week 0; §*P* < 0.05 when compared with control. ****P* < 0.001 refers to the comparison between IgG4- and mock-depleted paired samples. SMS = Combined symptom medication score; FEV_1_ = Forced Expiratory Volume in one second.

FIGURE S2 Time course of sIgG4, BAT, and BAT inhibition assay in Medication Group. Basophil CD63 responses to 0.15 and 15 μg/mL of Der p extract in Medication Group during the time course of BAT **(A)**. Time course of Der p sIgG4 in Medication Group **(B)**. The BAT inhibition assay was performed as serum from Medication Group patients incubated with 0.15 μg/mL (C; *n* = 35) or 15 μg/mL (D; *n* = 10) Der p allergen before performance of the BAT. Der p = *Dermatophagoides pteronyssinus*.

## Abbreviations

AHR: airway hyperresponsiveness
AIT: allergen immunotherapy
BAT: basophil activation test
Der f: *Dermatophagoides farina*
Der p: *Dermatophagoides pteronyssinus*
FEV_1_: forced expiratory volume in 1 s
FVC: forced vital capacity
SCIT: subcutaneous allergen immunotherapy
sIgE: specific immunoglobulin E
sIgG4: specific immunoglobulin G4
SMS: combined symptom medication score.
