# Supplementary figures and images for: Allergen Immunotherapy–Induced Immunoglobulin G4 Reduces Basophil Activation in House Dust Mite–Allergic Asthma Patients
Source: Front Cell Dev Biol. 2020 Feb 20;8:30. doi: 10.3389/fcell.2020.00030 (PMC7044416; doi:10.3389/fcell.2020.00030)

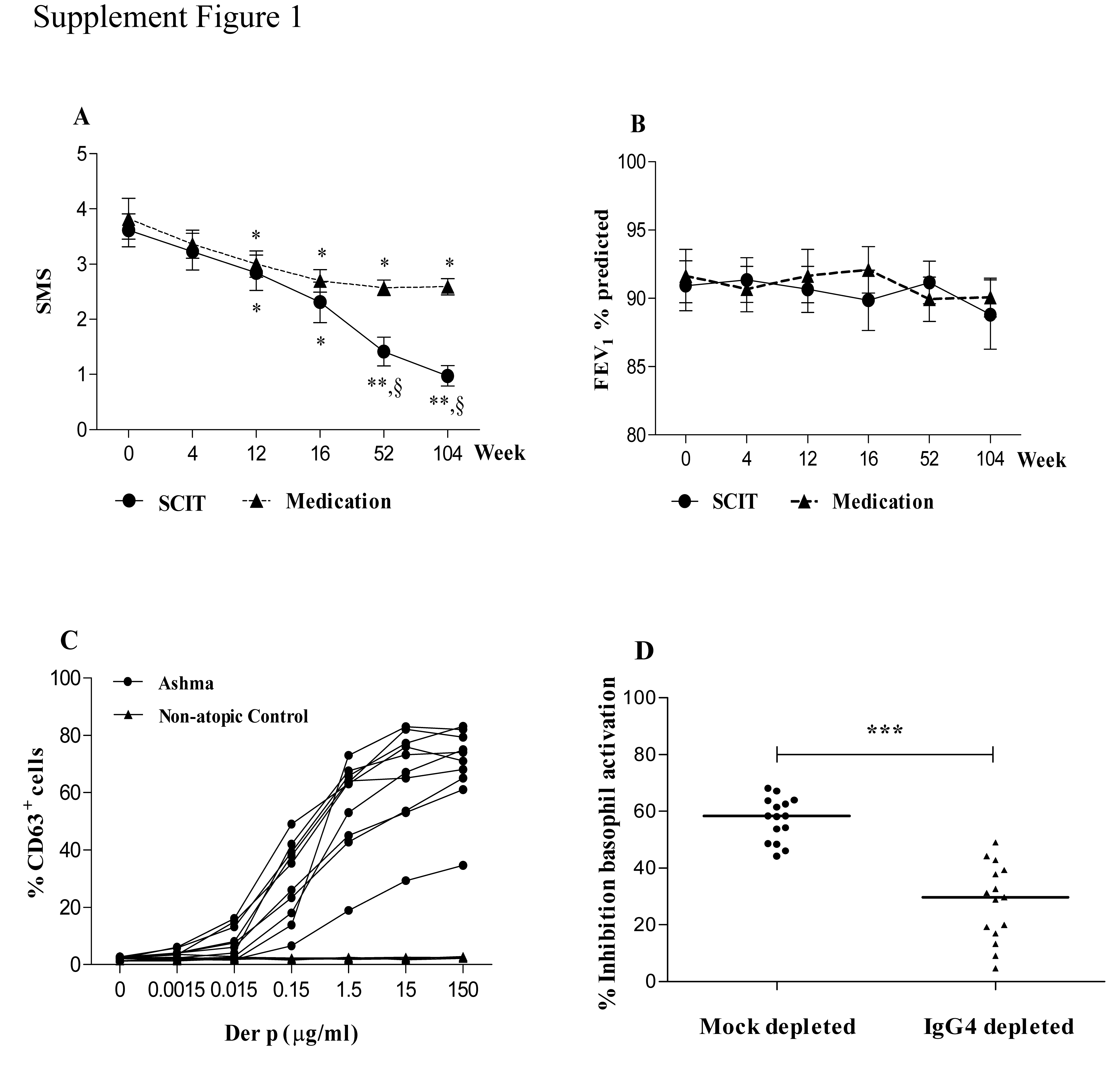

Supplement: FIGURE S1 — SMS and FEV1% over time. Allergen concentration curves for BAT, and IgG4 depletion BAT inhibition assay. Time course of mean SMS (A) and FEV1% predicted (B) in the SCIT group and medication group. Der p induced basophil activation in blood samples from allergic patients with mite allergy (n = 10), and non-atopic controls (n = 5) were assayed with six mite extract concentrations. Results are expressed as percentages of CD63+ basophils above basal values (C). Fifteen serum samples from SCIT patients at week 104 underwent removal of IgG4 antibodies. IgG4-depleted and mock-depleted samples were incubated with 0.15 μg/mL Der p allergen before performance of the BAT. *P < 0.05, **P < 0.01 when compared with week 0; §P < 0.05 when compared with control. ***P < 0.001 refers to the comparison between IgG4- and mock-depleted paired samples. SMS = Combined symptom medication score; FEV1 = Forced Expiratory Volume in one second. [file Image_1.TIF]

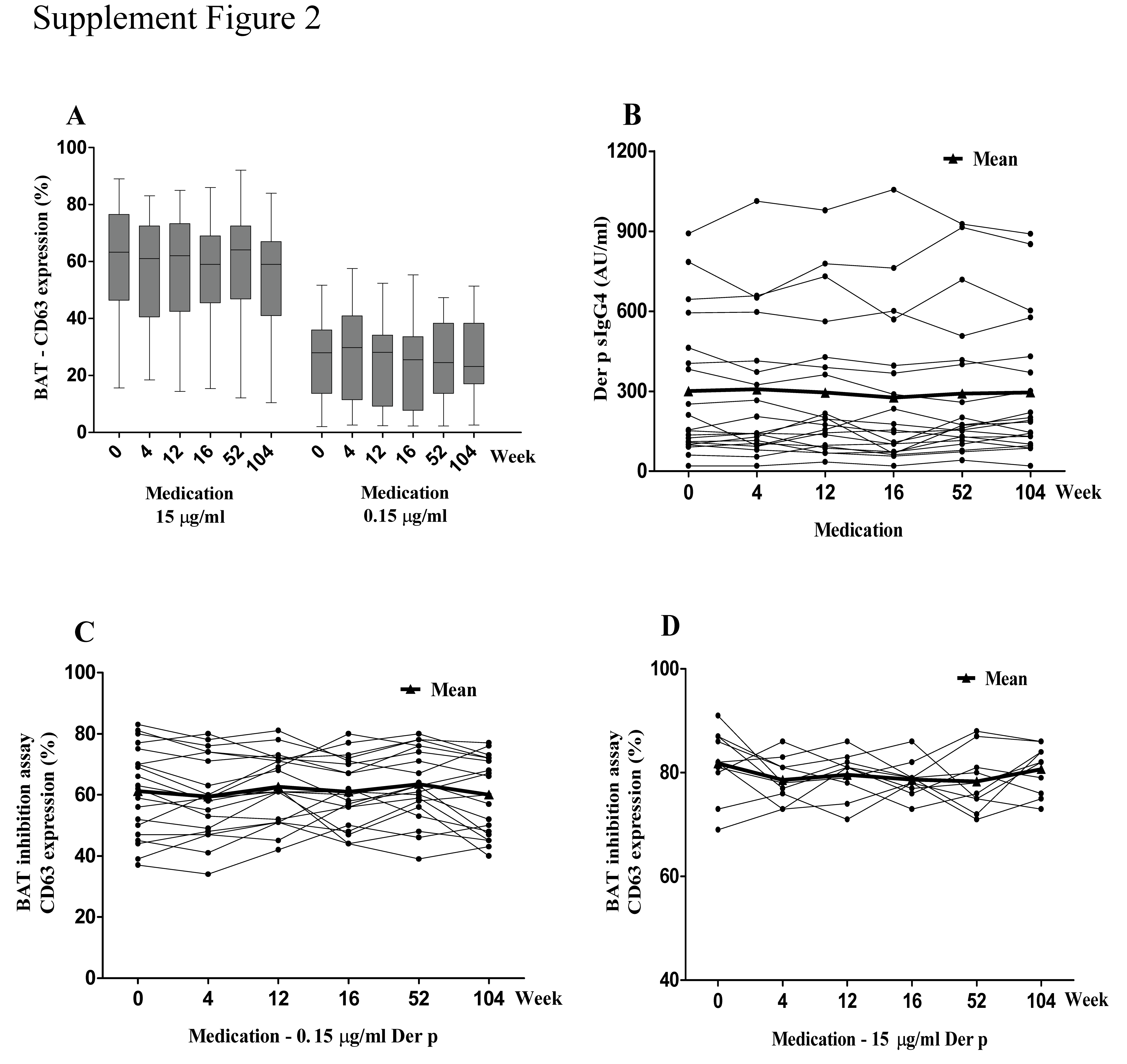

Supplement: FIGURE S2 — Time course of sIgG4, BAT, and BAT inhibition assay in Medication Group. Basophil CD63 responses to 0.15 and 15 μg/mL of Der p extract in Medication Group during the time course of BAT (A). Time course of Der p sIgG4 in Medication Group (B). The BAT inhibition assay was performed as serum from Medication Group patients incubated with 0.15 μg/mL (C; n = 35) or 15 μg/mL (D; n = 10) Der p allergen before performance of the BAT. Der p = Dermatophagoides pteronyssinus. [file Image_2.TIF]
